# Granular piston-probing in microgravity: powder compression, from densification to jamming

**DOI:** 10.1038/s41526-022-00235-2

**Published:** 2022-11-05

**Authors:** Olfa D’Angelo, Anabelle Horb, Aidan Cowley, Matthias Sperl, W. Till Kranz

**Affiliations:** 1 Institut für Materialphysik im Weltraum, Deutsches Zentrum für Luft- und Raumfahrt (DLR), 51170 Köln, Germany; 2 Institute for Multiscale Simulation, Universität Erlangen-Nürnberg, Cauerstraße 3, 91058 Erlangen, Germany; 3 European Astronaut Centre (EAC), European Space Agency (ESA), 51170 Köln, Germany; 4 Institut für Theoretische Physik, Universität zu Köln, 50937 Köln, Germany; 5 Present Address: Omnidea, Lda. – Polypark, Nùcleo Empresarial da Arruda dos Vinhos, Estrada da Quinta de Matos 4, 2630-179 Arruda dos Vinhos, Portugal

**Keywords:** Soft materials, Aerospace engineering

## Abstract

The macroscopic response of granular solids is determined by the microscopic fabric of force chains, which, in turn, is intimately linked to the history of the solid. To query the influence of gravity on powder flow behavior, a granular material is subjected to compression by a piston in a closed container, on-ground and in microgravity (on parabolic flights). Results show that piston-probing densifies the packing, eventually leading to jamming of the material compressed by the piston, regardless of the gravitational environment. The onset of jamming is found to appear at lower packing fraction in microgravity (${\varphi }_{J}^{\mu -{{{\rm{g}}}}}=0.567\pm 0.014$) than on-ground (${\varphi }_{J}^{{{{\rm{gnd}}}}}=0.579\pm 0.014$). We interpret these findings as the manifestation of a granular fabric altered by the gravitational force field: in absence of a secondary load (due to gravitational acceleration) to stimulate reorganization in a different direction to the major compression stress, the particles’ configuration becomes stable at lower density, as the particles have no external drive to promote reorganization into a denser packing. This is coupled with a change in interparticular force balance which takes place under low gravity, as cohesive interactions become predominant. We propose a combination of microscopic and continuum arguments to rationalize our results.

## Introduction

As humans make their way into space and plan to travel to sand-covered celestial bodies, the influence of (reduced) gravity on granular phenomena becomes a matter of concern^1^, and new strategies are sought to handle granular materials efficiently in various gravitational environments^2–5^. While the action of gravity is intuitively expected to weaken as objects become smaller, the collective behavior of small particles composing granular systems is significantly modified by the gravitational field, challenging our expectation of powder response outside of Earth’s gravity. Granular media are by definition composed of grains *big enough* not to be subject to thermal motion^6,7^ but *small enough* for continuous material properties to emerge on the mesoscopic scale. Such grains are, therefore, generally sufficiently large for gravitational forces on each of them not to be negligible in comparison to the other forces in present. The flow behavior of granular matter can thus change dramatically with a change in gravitational acceleration, such as that affecting a body on a foreign celestial surface (e.g. Moon, Mars, or an asteroid), or subjected to microgravity conditions.

The aim of this article is to investigate the densification of a granular packing in two scenarios: on-ground (gnd), where Earth’s gravity may accelerate particles relative to the fixed container, and in microgravity (*μ*-g), on parabolic flights, where gravity cannot induce such relative acceleration. Note, that the term *microgravity* is widely used, although *free-fall* would be more precise^8^.

**Fig. 1:**
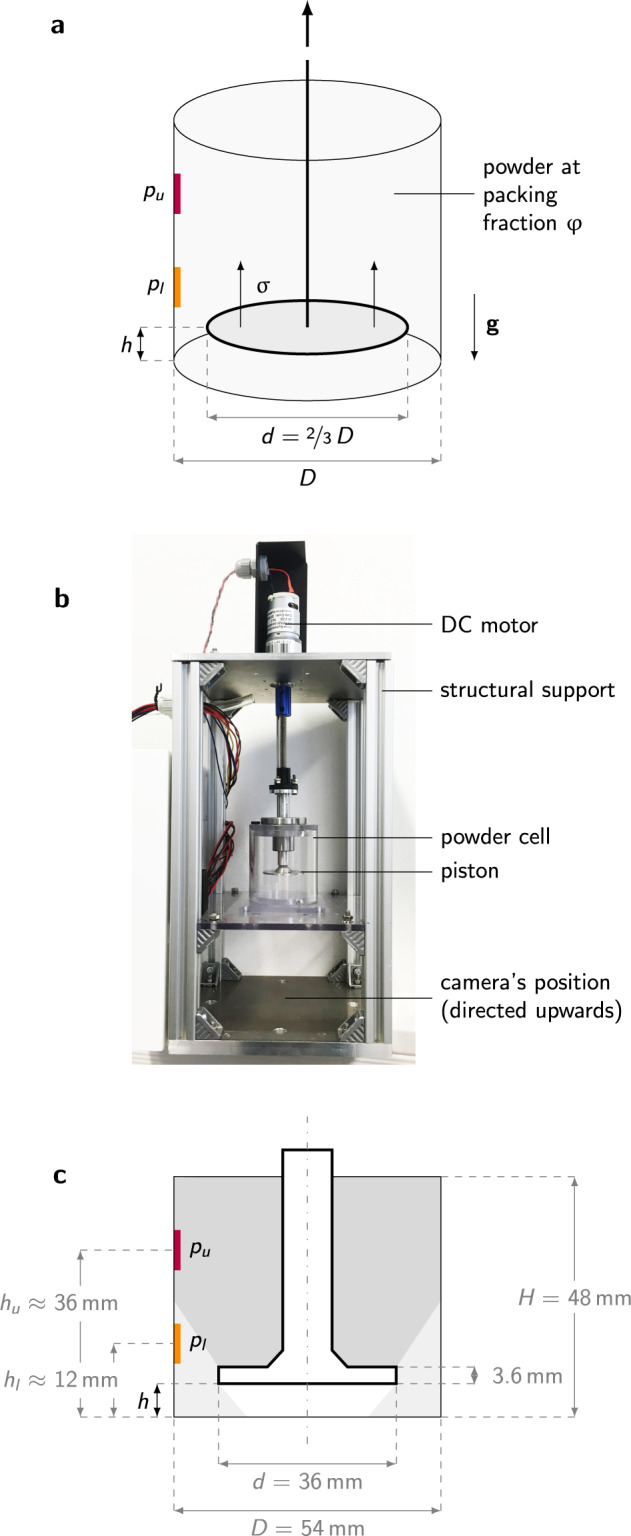
Piston-probing setup. **a** Piston-probing principle: a piston rises incrementally in a closed container, filled with powder at packing fraction *φ*, applying a normal stress ***σ*** on the granular packing. The pressure *p* transmitted through the granular material is recorded by two pressure sensors placed on the lateral walls of the container (respectively recording *p*_*u*_ and *p*_*l*_, *upper* and *lower* pressures). The direction of the gravitational acceleration *g* is indicated by a downwards-pointing arrow. **b** Experimental setup, showing the container (here empty) in its support structure (photograph taken by the authors). **c** Schematic including container’s and piston’s dimensions (in gray), and variables recorded (in black).

We propose a densification mechanism in the form of a piston rising quasi-statically through a confined powder bed (see Fig. 1), thereby compressing the material placed above. The piston is of diameter inferior to that of the container. Compression is continued until reaching a jammed state above the piston, hence probing the jamming point variations between the two environments. The jammed state is characterized by the appearance of a finite elastic modulus of the granular packing, which drives the piston to a stop. Such piston mechanism could be used, on-ground or in space, to densify shallow granular beds and probe the percolation of the force network, possibly before submitting the material to further processing.

In presence of gravity, granular flows can be caused by their potential energy in gravity-driven flows, providing a granular time scale in the form of the time needed for grain to fall by its diameter. In microgravity, particles do not fall towards the minimum-height position available; instead, they cluster into floating aggregates^9–12^, as the inter-particle cohesive forces become the predominant interaction. If no force field sediments the packing, particles or clusters are not necessarily in contact with their closest neighbors^13^. Force chains^14,15^, enabling force transmission through lasting contacts in a dense granular medium, cannot appear unless a contact network is established. Yet, such contact network can also trigger flow arrest on the macroscopic scale, if it percolates into a force network spanning the system, transmitting force to its boundaries akin to a solid^16–18^.

The jamming transition^19,20^ describes the changeover between the flowing state, in which the granular fabric deforms plastically, and the jammed state, where a mechanically stable contact network is formed, which can withstand a certain amount of stress without reorganizing. In other words, the granular assembly as a whole exhibits a finite elastic modulus. If unpredicted, the appearance of jammed regions can be detrimental to industrial processes involving handling and transport of granular materials, as it can draw entire production processes to a halt. In sheared granular media, an anisotropic shear-jammed phase can block powder flow mono-directionally^21–24^. It was suggested that for frictionless particles, this transition already occurs at random loose packing (rlp)^25,26^, while a granulate in the state of random close packing would be isotropically jammed^27,28^. However, the existence of a *fixed* jamming packing fraction *φ*_J_ (jamming point) has now been challenged^29,30^.

Besides evident dependence on particles’ shape^31,32^, (poly)dispersity^33^ or mechanical properties^32^, experimental, and numerical studies have shown the history-dependence of *φ*_J_, with variations, even for a single granular material, related to the preparation protocol^29–31,34,35^ or compression rate of the granular bed leading to jamming (namely, *φ*_J_ decreases with increasing compression rate^33,36^). Interparticular friction was shown to decrease the jamming packing by decreasing particles’ mobility in dense packing^22,32,37,38^.

It has been reported that when gravitational forces are negligible, the density of a granulate in random loose packing decreases from ${\varphi }_{{{{\rm{rlp}}}}}^{{{{\rm{gnd}}}}}=0.60$ to ${\varphi }_{{{{\rm{rlp}}}}}^{\mu -{{{\rm{g}}}}}=0.55$^25,39–42^ (for a granular material composed of hard monodispersed spherical particles with low or no friction in three dimensions). However, to the best of our knowledge, the impact of gravitational acceleration—and particularly of very low gravity ~ *μ*-g—on the packing fraction at the jamming transition has hitherto not been studied experimentally.

Before reporting on the actual measurements, let us make a naive prediction. We consider a piston placed at the bottom of a container filled with granular material at a density *φ* ≈ 0.55. As in our setup, the piston has a diameter *d* = 2/3*D*, where *D* is the container diameter. When the piston rises by a small height Δ*h*, it displaces the volume *πd*^2^Δ*h*/4 of powder above the piston. This may either be achieved by compacting the powder to a higher packing fraction above the piston, or by pushing the powder beneath the piston. On-ground, the material underneath the piston will settle in an annular heap at its angle of repose *α*. At small piston displacements *h*, the volume of that heap grows more slowly by a factor $2h/d\tan \alpha \ll 1$ compared to the displaced material on the top. Consequently, we expect the piston to stop shortly after the material is fully compacted. Under microgravity, however, the displaced material does not have to settle in a heap; it may instead fill the entire volume freed below the piston. Consequently, one may expect the piston to be able to rise indefinitely without being stopped by a jammed phase.

## Results

### Piston probing

Piston-probing consists of applying a finite amount of normal stress to a granular material in a confined space, by raising a piston inside a container filled with this material. Lateral space is left on the sides of the piston’s platform, allowing the granulate to yield and flow downwards, around the platform. If applicable, the gravity vector points downward, against the rising motion of the piston.

The experimental setup is presented in Fig. 1. It consists of a transparent PMMA tube of diameter *D* = 54 mm and height *H* = 48 mm, inside which a piston of diameter *d* = 36 mm can rise incrementally. The piston is moved by a direct-current (DC) motor, driving a traveling-nut linear translation stage that allows the piston to rise vertically without rotating. The translation stage can be placed in one of two modes by the user: (i) a stationary mode, where the piston is actively stabilized at its current position; and (ii) a traveling mode where the piston is actively pulling upwards. The DC motor is regulated through a velocity-controlled closed control loop that keeps the velocity at its set value until the resistance of the packing reaches a significant fraction of the motor’s maximum torque, at which point the piston slows down and eventually stops. The maximum torque was chosen such, that the experiment probes the elastic modulus of the jammed packing, yet only minimally deform the particles which are much stiffer (see Methods). Optical measurement of the piston height *h* provides an indirect assessment of its position, thus of its true rising speed.

Pressure sensors are placed inside the tube, on the lateral walls, and record the pressure evolution as the piston compresses the granular sample. One sensor is placed on the upper part of the container (*h*_*u*_ ≈ 36 mm), and another one on the bottom part (*h*_*l*_ ≈ 12 mm). The sensitive area of the pressure sensors measures 200 mm^2^, which represents ~30,000 particles. The pressure sensors are calibrated in-house (see Methods for details), so the pressure recorded corresponds to the pressure difference due to the powder contained in the cell. A camera below the transparent container allows observing inside the setup from below. At the beginning of the experiment, the piston is generally at its lowest position, i.e., the platform touches the bottom of the closed tube; it is then programmed to rise step-by-step, each step being activated by the user during ~20 s. In some experiments, the platform is placed in the middle of the box, at its median position in height (*cf*. results shown in Fig. 4a, b). Hardware technical specifications are given in Table 1, and a list of all experiments reported is provided in Supplementary Material (Supplementary Table 1).

**Table 1:** References for hardware used on the piston-probing experimental setup.

Hardware	Model
DC motor	DSMP 320-24-0189-BF
	(max. torque 0.69 Nm)
Current sensor	Pololu ACS7174
Pressure sensor	SingleTact capacitive pressure sensor
Camera	YI 4K Plus Action Camera
	(resolution 3840 × 2160 pixels)

The granular material used to fill the container is a monodispersed polystyrene (PS) powder with spherical particles of diameter 80 *μ*m (see Methods for further details). Inside the closed container, the mass of granular material inserted determines an overall packing fraction of *φ*_0_ ≈ 0.55. At the beginning of the experiment (that is, until reaching a jammed state), the packing fraction is assumed to be sufficiently homogeneous to be calculated from this initial packing fraction *φ*_0_ and the piston’s position in height, *h*. Therefore, the packing fractions given throughout this article, and in particular the packing fractions at the jamming transition *φ*_J_, are *global* packing fractions; the local packing fractions are not determined experimentally. A calculation of formal uncertainty is provided in the Methods section.

Let us consider the most common case, where the piston is initially at the bottom of the container (*h* = 0). The piston diameter being two thirds of the container, a lateral space is left around the piston, through which the granular material can flow downward to reach the bottom of the container (below the piston). The dimensions of the piston are chosen such, that the upward flow imposed by the piston rise could be accommodated by a downward flow through the gap around the piston. Maximum speed and force of the piston were chosen to give the packing ample time to relax, yet allow for significant piston motion within the limited time intervals of microgravity, and to limit particle deformations in the jammed state.

During the initial instants of the experiment, the powder distribution under the piston can be observed through a transparent bottom plate. At all stages of the experiment, the piston position *h* and the pressure on the outer walls at two heights, *p*_*u*_ and *p*_*l*_ (respectively *upper* and *lower*), are recorded.

This experiment flew on the 31st DLR parabolic flight campaign (PFC) in March 2018. PFCs provide periods of microgravity (~10^−3^*g*) of 22 s as the plane (and its content) is in free-fall, alternating with hypergravity (≈1.8 *g*) phases (as the plane rises and swoops). During the free-fall period, in the frame of the plane, the directional force of gravity is replaced by approximately isotropic, residual accelerations (termed *g*-jitter) on the level of ~ 0.05 *g*. Here *g* = 9.81 ms^−2^ denotes the standard acceleration of gravity. For an overview of parabolic flights as a gravity-related experimental platform, the reader is referred to ref. ^8^.

Experiments conducted on parabolic flights are reproduced on-ground, using the same experimental setup in the laboratory. It should be noted that the flight time up to the first parabola, as well as the first hypergravity phase might contribute to create an initial state in microgravity that differs from their on-ground counterpart.

A two dimensional (2D) toy model reproducing a vertical section of our setup with photoelastic particles^43,44^ is used to help visualizing the dependence of the force chains network on a secondary force field acting downwards (see Supplementary Material, Supplementary Figure 1).

### Time evolution of pressure and density

**Fig. 2:**
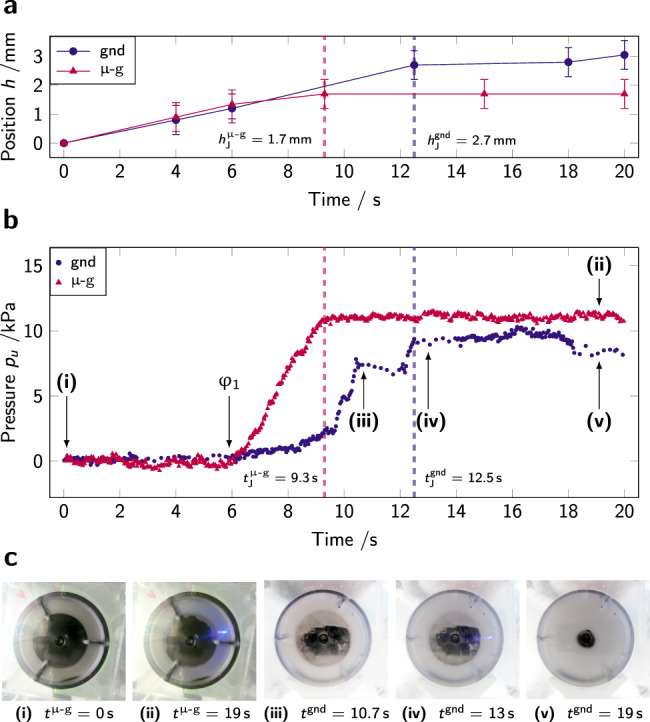
Time evolution of piston position and pressure in powder cell. **a** Piston position in height as a function of time. Error bars represent the experimental precision in optical measurement. **b** Pressure recorded by the top sensor during the first step up of the piston, on-ground (blue disks, gnd), and in microgravity (red triangles, *μ*-g). The jamming times *t*_J_ are marked by vertical dashed lines, as well as *φ*_1_ (see the body of the text) and five points of interest that correspond to **c**, images (i–v). (i) and (ii) are images from the bottom of the experiment cell looking up in microgravity (snapshots taken by the authors); (iii), (iv), and (v) on-ground. The piston is black and the PS powder, white. The clearly visible piston at all times in microgravity (i, ii, iii) indicates the absence of powder flow below the piston, different from the behavior on-ground (iv, v).

Pressure data from the first step up of the piston, measured on the upper half of the container, on-ground (gnd) and in microgravity (*μ*-g), is presented in Fig. 2. For all microgravity experiments, *t* = 0 s corresponds to the start of the microgravity period. For experiments conducted on-ground, it corresponds to the start of the piston rise.

**Fig. 3:**
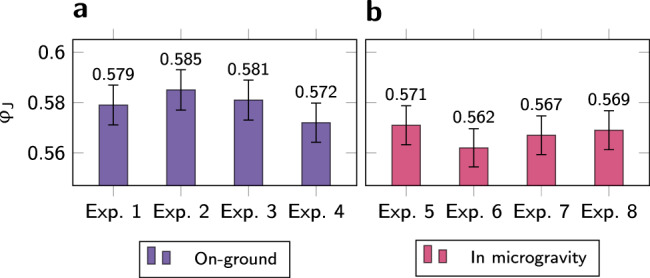
Packing fraction at jamming *φ*_J_, estimated from the height at which the piston is driven to a halt by the resistance of the packing. Experiments are conducted in the same setup, **a** on-ground (gnd) and **b** in microgravity (*μ*-g). Error bars represent the formal accuracy (see Methods section for details on the calculation).

At first and until approximately 6 s, the piston is rising (Fig. 2a) but the pressure does not undergo any change (Fig. 2b). In this initial phase, the powder is compressed from the initial packing fraction *φ*_0_ to a denser configuration, *φ*_1_ > *φ*_0_. For both experiments, only then does the pressure started increasing. A second characteristic density, *φ*_J_, is reached when the piston is brought to a halt and the powder resists the maximum stress imposed on the sample. By recording the piston’s position at this instant, global estimates of *φ*_*J*_ have been obtained in four repetitions of the experiment, each on-ground and under microgravity conditions; resulting *φ*_J_ for each repetition are presented in Fig. 3. Note that the jamming density is systematically lower in microgravity compared to ground. Statistical significance test shows that albeit the small number of repetitions available, this finding is statistically significant (see Methods section).

**Fig. 4:**
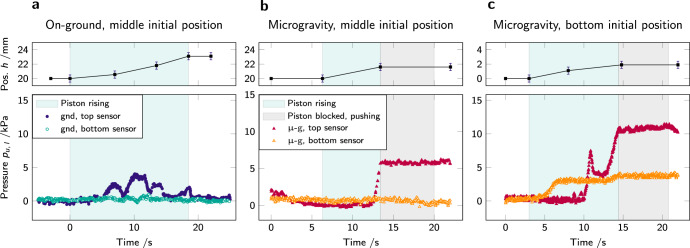
Pressure evolution with piston rise. **a** (gnd) and **b**, **c** in microgravity (*μ*-g). The pressure *p*_*u*, *l*_ (bottom graphs) is recorded by sensors in two positions: on the top part of the cell (*p*_*u*_: filled marks, gnd in blue and *μ*-g in red) and on the bottom part of the cell (*p*_*l*_: open marks, gnd in green and *μ*-g in orange). On **a**, **b** the piston starts in the middle of the cell (position *h* ≈ 20 mm), while on **c** it starts at the bottom of the cell (*h* = 0 mm): on **a** and **b** the pressure sensors are distributed one above the piston and one below, while on **c** both sensors are above the piston. On **a** *t* = 0 s represents the start of piston rise; on **b**, **c**, *t* = 0 s represents the start of the microgravity phase. The pressure evolution exhibits repeated yielding on-ground, while the packing jammed in microgravity remains stable, even at maximum load. In the top graphs, the error bars represent the experimental precision in optical measurements.

**Fig. 5:**
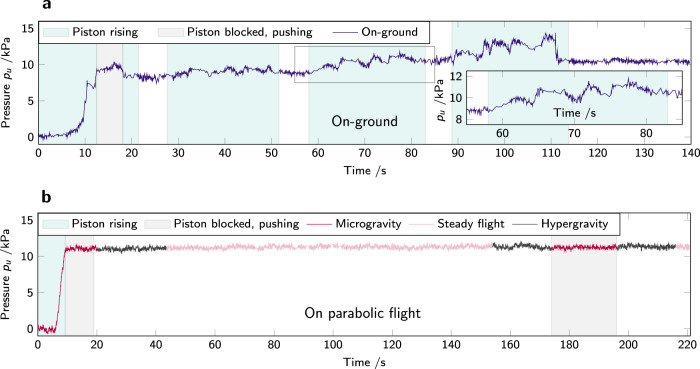
Pressure evolution for a duration of ≈200 s after the initial densification step leading to jamming. Pressure is recorded by the top sensor inside the powder container. **a** on-ground (gnd) and **b** microgravity (*μ*-g). The inset of **a** is a close-up of the pressure evolution during the second step-up of the piston, where the time and pressure range expanded in the inset are highlighted on the main panel by a gray rectangle. After an initial steep rise in pressure both on-ground and in microgravity, corresponding to jamming of the granular packing, on-ground the packing yields under the stress applied by the piston rise. On the contrary, the packing jammed in microgravity remains stable against hypergravity and against pressure applied by the piston in the next microgravity period.

Above *φ*_1_, the behavior of the packing depends on the gravitational environment. On-ground, the powder undergoes a first large-scale reorganization at *t*^gnd^ = 10.5 s (point (iii) in Fig. 2) which temporarily releases pressure on the sensor, but with almost no powder flowing under the platform, as seen in Fig. 2c images (iii) and (iv). The jamming packing fraction ${\varphi }_{{{{\rm{J}}}}}^{{{{\rm{gnd}}}}}$ is reached at ${t}_{{{{\rm{J}}}}}^{{{{\rm{gnd}}}}}=$ 12.5 s. The position of the piston in height correlates with the pressure measured on the wall of the container: the piston is blocked and stops rising when a peak in pressure is reached. At this point, the powder enters a creep regime of slow deformation and eventually yields at *t*^gnd^ = 18 s. As a result, material flows under the platform, releasing the pressure on the container wall, as seen in Fig. 2c, image (v). By placing the piston higher in the tube, yielding can be enhanced to the point that it repeatedly relaxes the pressure completely and allows the piston to continue to rise (*cf*. Fig. 4a). Apart from small dynamic excursions as a material is flowing down, the pressure on the wall below the piston is unaffected by the developments above the piston. The creep response may extend over minutes (*cf*. Fig. 5) with smaller pressure variations—indicative of continued internal rearrangements—interspersed by yielding events that deposit more material beneath the piston.

In microgravity, the granular behavior is quite different. Approximately at the same time as for the ground experiment (*t*^*μ*−g^ = 6 s), the pressure rises on the sensor; however it is not "jerky" as on-ground, but a smooth and relatively steep increase towards its saturation pressure, indicating an effective elastic response of the packing. The maximum pressure is reached earlier than on-ground: at ${t}_{J}^{\mu -{{{\rm{g}}}}}=$ 9.3 s. It should also be noted that at this instant (point (ii) in Fig. 2), despite the platform having risen by 1.7 mm, almost no powder has flowed under the platform: only the fringe of the piston’s platform is slightly covered by <1 mm of (white) powder. This is attributed to gravity jitter in the horizontal plane, common on parabolic flights^45^.

In Fig. 4, pressure evolution is presented for one step up of the piston, on-ground (4a) and in microgravity (4b, c). The pressure *p* is recorded on the top and bottom part of the container (respectively *p*_*u*_ and *p*_*l*_). In Fig. 4a, b, at *t* = 0 s the piston is in the middle of the cell: the top sensor records the pressure above the piston and the bottom sensor, that under it. In particular, for the experiment shown in Fig. 4b, the piston, placed at half height of the cell, is its initial position. In Fig. 4c, the initial piston position is at the bottom of the cell, hence both sensors are placed above the piston.

While placing the piston higher in the tube does not facilitate yielding as it does on-ground, occasionally, large-scale rearrangements can be observed in the initial buildup of pressure (see respectively Fig. 4b, c). The latter Fig. 4c also shows that densification proceeds inhomogeneously throughout the sample. We observe that until *t* = 10 s, the upper sensor does not record any substantial signal change, while the lower one already undergoes a pressure increase of ~3 Pa from *t* = 5 s, as the piston starts to rise in the container. Our conjecture is that at a point where the lower pressure sensor already registers substantial stresses, no force is yet transmitted to the upper part of the packing, as the contact network is not yet formed.

**Fig. 6:**
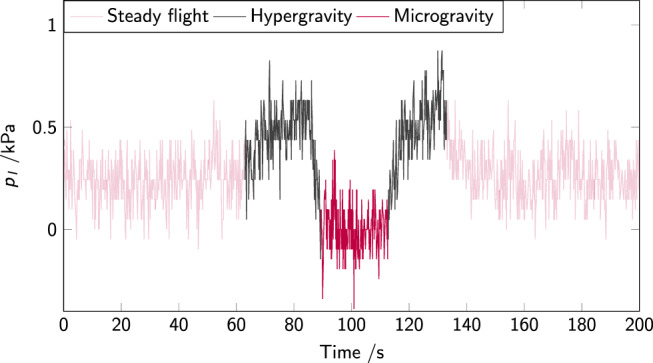
Pressure *p*_*l*_ on the bottom sensor inside a cell filled with powder, but where the piston does not move: the pressure changes are solely due to changes in acceleration during the parabolic flight maneuvers. Initial time *t* = 0 s is not a significant instant.

In Fig. 5, the pressure is observed for a longer duration after the first step up of the piston, on-ground (Fig. 5a) and in microgravity (Fig. 5b). On-ground, four repetitions of the piston rise lead to pressure buildup followed by collapse after 5 s. The inset is a close-up of the third step-up of the piston. Remarkably, there is no noticeable creep in microgravity. The recorded pressure does not change through repeated microgravity and hypergravity phases. In order to verify that this is not due to malfunction of the pressure sensors, on a different flight day the pressure was recorded during multiple parabolas without any motion of the piston (remaining in its initial position at the bottom of the cell). The result is presented in Fig. 6. If the packing is left in its initial unjammed state (no compression applied), the pressure variations due to change in gravitational acceleration are clearly visible, and corresponds to the temporal structure of the parabolic flight: hypergravity–low gravity–hypergravity, surrounded by steady flight. (The reader is referred to Fig. 9 for a longer pressure measurement without piston motion).

Note that all the experiments reported in Figs. 2–5 involve the same maximum piston load. However, the corresponding pressure on the side walls depends on the distance between pressure sensor and piston. In particular, the pressure increases with the distance to the piston.

To summarize, the granular sample subjected to piston-probing undergoes the following steps:

1. Densification of the granular medium: the packing density increases on top of the piston, leaving an empty volume under it.
2. The granular medium reaches a critical packing fraction (*φ*_1_) above the piston, dense enough to transmit forces from the rising piston to the sensors on the container walls. Pressure on the side walls increases and the piston slows down.
3. In microgravity, the pressure increases continuously until the piston stops at maximum load. We label this point "jamming point". Note that this state is stable under hypergravity as long as the piston is not lowered.
4. On-ground, the pressure also increases until a maximum load is reached and the piston stops. Again, it is this first stop of the piston that we label "jamming point". Eventually, the packing yields and flows into the empty space below the piston.

### Identification of regions in the packing

The piston-probing experiment conducted on-ground and in microgravity reveals a gravity-dependent response of the granular material on two main aspects:

- Under compression, jamming occurs in microgravity at lower packing fraction than on-ground.
- The packing eventually yields and flows on-ground, due to the secondary (gravitational) force field; in its absence, i.e. in microgravity, the granular material arranges into a highly stable jammed state.

**Fig. 7:**
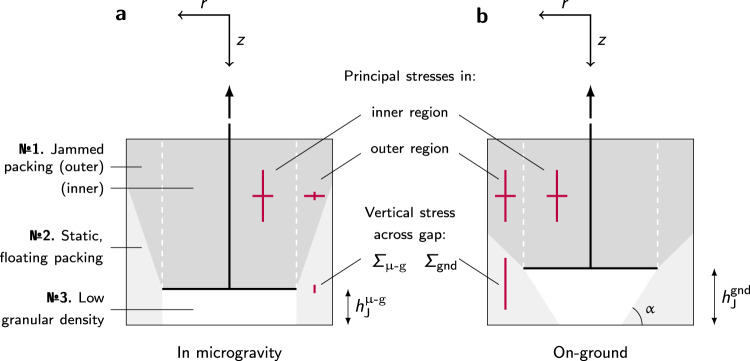
Schematic representation of the regions appearing in the container as the piston rises. **a** in microgravity (*μ*-g) and **b** on-ground (gnd). On-ground, the powder forms a slope at its angle of repose *α* with the horizontal. Stress magnitudes in arbitrary units are depicted by red lines. The coordinate system used in the body of the text is shown above each schematic. The height ${h}_{J}^{\mu -{{{\rm{g}}}},\,{{{\rm{gnd}}}}}$ denotes the height at which the piston is stopped by the jammed material above it, respectively under microgravity and on-ground conditions. The difference in force chain configuration between microgravity and ground leads to drastically different vertical stresses in the outer region. The load Σ on the material below the piston is, as a consequence, much lower in microgravity compared to the ground. See also Supplementary Material, Supplementary Figure 1.

To understand the granular physics behind the observed behavior and explain the gravity dependence, we need to have a closer look at the granular material in the apparatus. To this end, it is helpful to conceptually distinguish three different regions inside the tube, represented in Fig. 7. Region №1 is the material above the piston, compacted as the piston rises; it is the region where the granular material eventually jams. Region №3 is the region below the piston, left empty as the piston rises. In between, region №2 is the region around the piston, which can flow into the empty region №3.

The task ahead can therefore be structured into: (1) understanding the compaction phase of region №1 and the resulting granular fabric; (2) understanding the stress distribution in region №1 that determines the force on region №2; and, finally, (3), understanding the yield criteria of region №2 in the presence of an empty region №3.

### Compaction of region №1

While characterizing the point of transition to jamming is still a controversial topic^20,30,39,46,47^, the mesoscopic scale phenomenon at its origin is generally accepted: compression-induced jamming occurs if strong force chains appear along the major compression stress direction, in response to the load itself^48^. The presence or absence of a secondary (gravitational) force field modifies the force distribution in the different zones of the packing^49^.

At *φ*_0_, prepared under ground conditions, the packing assumes a configuration that is rigid against the unidirectional gravitational body force, as long as no other load is applied. This structure, however, is no longer rigid against the unidirectional boundary force applied by the piston, and the packing can initially be compacted with negligible force.

Due to frictional interactions, vertical forces are partially supported by the vertical outer walls of the tube. Force chains, consequently, arch in the direction of the net force. When the latter changes from the downward orientation due to gravity, to the upward orientation imposed by the piston, we can infer that the force chains change curvature. Where the forces balance, the force chains buckle. For cohesionless particles, this would in itself allow the packing to further collapse under the influence of gravity as the unloaded contacts between particles are no longer constrained by friction and/or cohesive interactions.

At this point it is instructive to estimate the maximal stress imposed by gravity on-ground. From the analysis proposed by Janssen^50^, we find a screening length *ℓ* ≈ 26 mm, which limits the gravitational stress even at maximum depth *H* to *σ*_*g*_ ≈ 125 Pa (*cf*. Eq. (8)). As seen below, the cohesive energy density is at least on the order of 10 kJ m^−3^, (equivalent to 10 kPa, as stress and energy density have the same units). Typical forces during buckling of the force chains will therefore be unable to break most contacts. The new network of force chains will initially form by mostly establishing additional contacts between particles, which necessarily implies a densification of the packing.

As a result of the densification, *φ*_1_ is eventually reached. We can infer the following mechanism on the microscopic scale: the granular fabric supports load-bearing force chains again, as the chains of contacts above the piston have percolated into a network transmitting the load imposed by the piston to the container’s walls. However, this network is not stable against the piston-imposed load: the particles can still reorganize into denser configurations.

As the piston does not cover the whole cross-section of the tube, we may conceptually distinguish between an *inner* region №1 and an *outer* region №1. The inner region directly transmits the forces of the piston, while the outer region is indirectly affected through the horizontal redistribution of stresses in the packing. Under gravity, as soon as the stress due to the piston rise exceeds the gravitational stress, forces are directed upwards while the outer region may still be forced down by gravity. This creates a shear force between inner and outer that intermittently severs force chains. The resulting local force balance may lead to large-scale rearrangements that are at the origin of the pressure drops observed on-ground between *φ*_1_ and *φ*_J_. Note that the stresses imposed by the piston considerably exceed the gravitational stresses in the inner region for most of the compression phase such that gravity is negligible there.

At *φ*_J_, the packing has found a configuration that is stable against the maximal piston force with respect to local rearrangements of particles. On longer time scales, however, for the experiments conducted on-ground, the evolving pressure signal indicates an ongoing evolution of the force chain network under constant load (see Fig. 5a). Note that the pressure on the side walls is increasing over time, which implies that the deflection of forces away from the vertical also increases and the vertical normal stress decreases in the upper parts of the piston. This aging or creep phenomenon can be rationalized in terms of soft glassy rheology (SGR)^51,52^ and its adaption to jammed packings^53^. On a continuum level, force chains entail a spatially heterogeneous stress distribution. Global stress relaxations may therefore occur by chains of local relaxations that proceed at a finite speed. In the framework of Edward’s ensemble of granular packing, the *angoricity* is the intensive thermodynamic parameter conjugate to the stress; it quantifies the sensitivity of the stress heterogeneities to the imposed load^54^. Within SGR, jamming occurs if the angoricity *β* falls below a critical value *β*_*J*_. In the jammed state, *β* < *β*_*J*_, SGR predicts aging^52^, compatible with our measurements.

For the microgravity experiments, upon entering the microgravity phase of each parabola, the cohesive forces among particles become predominant^12^ and create a packing where once particles come into contact with their closest neigbours, those contacts are likely to remain. Upon compression and up to *φ*_1_, the packing collapses—like on-ground, mostly by local buckling and by preserving existing contacts – into a load-bearing configuration. However, no strong shear forces appear as the piston is the single source of stress on the mesoscopic scale. Note that friction with the piston’s wall is generically smaller than the internal friction of the granular packing^55^. At the slow speed of the piston, the wall friction therefore cannot generate shear forces between the inner and outer region. Remarkably, under these conditions, the topology of the force chain network seems to be independent of the magnitude of the imposed stress. As a result, the measured pressure *p*_*u*, *l*_ becomes proportional to the strain imposed by the piston and the packing behaves as an elastic solid with a well defined elastic modulus. As a rough estimate from Fig. 2, let’s assume a strain of 1% during the buildup of pressure from 0 kPa to 10 kPa. This yields an elastic modulus on the order of 1 MPa, compatible with theoretical predictions (see Methods Section).

On the time scale of the experiment, no creep can be observed in microgravity. In terms of angoricity, this implies a vanishing sensitivity to the magnitude of the load, *β*→0. This is in line with the elastic regime observed before for *φ* < *φ*_*J*_. At this stage, we can only deduce that a single boundary force can lead to *β* = 0 while the competition between said boundary force and the gravitational body force leads to a finite *β* > 0. We invite further studies that investigate this more systematically.

A 2D hands-on version of a section of this experiment using photoelastic discs allowed us to visualize the force chains distribution within the granular medium; it qualitatively confirms the appearance of the three regions sketched in Fig. 7. Results are shown in Supplementary Material (Supplementary Figure 1).

### Stress distribution in region №1

In order to understand the load the piston is indirectly enforcing on region №2, one has to understand the stress distribution in region №1. Of particular interest is the normal stress Σ imposed by region №1 to region №2 through the gap around the piston.

The observation that the vertical free surface of the granular material below the piston is unstable at heights of the order of millimeters if prepared on-ground, yet remains stable even under hypergravity conditions if prepared in microgravity, is perplexing, at first. It is a testament to the nontrivial stress distribution in a granular sample. In the following, we will propose a continuum argument to rationalize this observation.

Following Janssen^50,55^, one may, for the sake of the argument, assume that the vertical, Σ_*zz*_, and horizontal, Σ_*rr*_, normal stresses are the principal stresses. Unlike in a molecular fluid, the two normal stresses are not identical, even in the absence of gravity, and are not homogeneous throughout the material. Again following Janssen, we restrict our discussion to a two-dimensional, vertical cross-section of the experiment (which is in principle trivial to extend to 3D, given the axisymmetry of our experimental setup).

At finite gravity but with an immobile piston, the experiment is equivalent to the silo Janssen had in mind. We consider a silo with a circular cross-section of diameter *D*; in summary, the major principal stress is Σ_*zz*_ and the minor principal stress, Σ_*rr*_ = *k*Σ_*zz*_, is reduced by Janssen’s constant *k* < 1. Friction with the wall will screen the weight on a length scale *D*/4*μk*. Here, we focus on the radial rather than the vertical structure of the problem, and just keep in mind that from one end to the cylinder to the other, stresses will be renormalized by the screening effect.

Let’s begin by recalling that the normal stress due to gravity on the gap around the piston, Σ ≡ Σ_*zz*_(*z* = *H*)≤*σ*_*g*_ ≈ 125 Pa. Now we imagine a significant normal stress *σ* ≫ *σ*_*g*_ being applied by the piston, such that we can neglect gravitational stresses in the following. In addition, consider only the material directly above the piston, for *r* < *d*/2. The Janssen effect will screen the stresses on a length scale *ℓ* = *d*/4*μk*, leading to a depth-averaged vertical normal stress ${\overline{{{\Sigma }}}}_{zz}^{\,{ < }\,}={C}_{1}\sigma$ and related to this, an average horizontal normal stress ${\overline{{{\Sigma }}}}_{rr}^{\,{ < }\,}=k{\overline{{{\Sigma }}}}_{zz}^{\,{ < }\,}$. Here *C*_1_ = *C*_1_(*H*/*ℓ*) < 1 is a dimensionless constant encoding the geometry of the setup. From Fig. 4c we infer that the pressure between the two heights differs by about a factor 2.5 in the fully jammed state. Given the distance between the sensors (see Fig. 1), we obtain an estimate for the screening length, *ℓ* ≈ 26 mm.

If the packing is prepared on-ground, the major principal stress will also be vertical in the outer region, *D*/2 > *r* > *d*/2, but the geometry of the outer region will induce its own screening length *L* ≈ *D*. If we assume the applied stress to dominate over gravity, *σ* ≫ *σ*_*g*_, then in the outer region  ${\overline{{{\Sigma }}}}_{zz}^{ \,{ > }\,}={C}_{2}(L){\overline{{{\Sigma }}}}_{rr}^{\,{ < }\,}/k$, while ${\overline{{{\Sigma }}}}_{rr}^{ \,{ > }\,}(r)={\overline{{{\Sigma }}}}_{rr}^{\,{ < }\,}d/2r$. (Note that *C*_2_(*L*) = *C*_2_(*H*/ℓ, *H*/*L*, ℓ/*L*) < 1 is another dimensionless geometrical factor). In particular, the pressure recorded on the side wall $p\approx {\overline{{{\Sigma }}}}_{rr}^{ \,{ > }\,}(D/2)=({C}_{1}kd/D)\sigma$ and the stress on the gap ${{{\Sigma }}}_{{{{\rm{gnd}}}}}\approx {\overline{{{\Sigma }}}}_{zz}^{ \,{ > }\,}={C}_{2}(L){C}_{1}\sigma$. From this, we conclude Σ_gnd_ ≈ *p*/*k*. Up to renormalizations, the normal stress on the gap is essentially given by the stress applied through the piston. The pressure recorded on the side wall, on the other hand, is proportional to *kσ*.

However, if prior to applying a stress, the sample is prepared in microgravity conditions, the compaction in the outer region is effected by the horizontal normal stress, ${{{\Sigma }}}_{rr}^{\,{ < }\,}$, exerted by the inner region. Therefore the major principal stress is now horizontal instead of vertical in the outer region and only a fraction *k* is transmitted to the vertical direction, ${{{\Sigma }}}_{zz}^{ \,{ > }\,}=k{{{\Sigma }}}_{rr}^{ \,{ > }\,}$. This will shorten the screening length in the outer region to $L^{\prime} ={k}^{2}L$. For the average vertical normal stress, we now have ${\overline{{{\Sigma }}}}_{zz}^{ \,{ > }\,}=k{C}_{2}(L^{\prime} ){\overline{{{\Sigma }}}}_{rr}^{\,{ < }\,}$, i.e., ${{{\Sigma }}}_{\mu -{{{\rm{g}}}}}\approx {\overline{{{\Sigma }}}}_{zz}^{ \,{ > }\,}={k}^{2}{C}_{2}(L^{\prime} ){C}_{1}\sigma$, while the pressure on the side wall remains essentially unchanged. From this, we conclude Σ_*μ*−g_ ≈ *kp* and most importantly Σ_*μ*−g_/Σ_gnd_ ≈ *k*^2^ independent of the applied load.

Notably, the load on the packing below the piston is significantly reduced under microgravity conditions. The additional weight induced stress in a subsequent hypergravity phase is heavily screened by the short length scale $L^{\prime} \approx {k}^{2}D\approx 9\,{{{\rm{mm}}}}$, to roughly $\gamma L^{\prime} \lesssim$ 50 Pa. This is well within the stress fluctuations due to g-jitter. If the packing was stable to the latter, it will not yield in hypergravity either. As a rough estimate, we infer from the maximum *p* ≈ 11 kPa,

1a $${{{\Sigma }}}_{{{{\rm{gnd}}}}}\approx \sigma \approx \frac{p}{k\left(1-{e}^{-H^{\prime} /\ell }\right)}\approx 40\,{{{\rm{kPa}}}},$$

and

1b $${{{\Sigma }}}_{\mu -{{{\rm{g}}}}}\approx {k}^{2}\sigma \approx \frac{kp}{\left(1-{e}^{-H^{\prime} /\ell }\right)}\approx 6.5\,{{{\rm{kPa}}}},$$

where $H^{\prime} =30\,{{{\rm{mm}}}}$ is the approximate distance between the upper surface of the piston and the pressure sensor at maximum load.

Note that Mohr–Coulomb theory has nothing to say on the creep behavior. From the slowly growing horizontal stress at constant vertical load, we may infer that Janssen’s constant is also slowly growing in time and the packing evolves towards a slightly more isotropic stress distribution.

### Stability of region №2

First of all, note that a vertical free surface of a granular packing can only be stable due to cohesion, *c* > 0. Effective values for the cohesive stress (or energy density) are hard to come by and do not only depend on the particles but also on the preparation. However, typical values for ordinary materials are on the order of several kilopascal. Under gravity, the weight stress *γh* limits the maximum stable height of a vertical surface to ${h}_{\max }=4c/\gamma (\sqrt{1+{\mu }^{2}}-\mu )$^55^. With *γ*_gnd_ ≈ 6 kPa m^−1^, one obtains stable heights on the order of meters. It is obvious that we cannot expect collapse of the free surfaces of a couple millimeters in height that occur in the experiment even under hypergravity. In addition, we have seen above that the load imposed by the piston on the gap, even under unfavorable microgravity conditions, by far exceeds the gravitational load.

Mohr–Coulomb theory also provides the maximum load that can be sustained by a vertical wall, ${{{\Sigma }}}_{\max }=2c/(\sqrt{1+{\mu }^{2}}-\mu )$^55^. Given that the experiment yields on-ground but not in microgravity, we can deduce a cohesion strength on the order of a few kilopascal in line with expectations. Once region №2 yields underground conditions, the material will settle in a heap with the angle of repose *α* determined by the material properties. This limits the amount of material that can be deposited below the piston, as alluded to in the introduction.

### Shift of the density at jamming

Concomitantly to the maximum height reached by the piston under the different experimental environments, the critical packing fraction at which jamming occurs under compression is found to be lower in microgravity (on average ${\varphi }_{{{{\rm{J}}}}}^{\mu -{{{\rm{g}}}}}=0.567\pm 0.014$) than on-ground (on average ${\varphi }_{{{{\rm{J}}}}}^{{{{\rm{gnd}}}}}=0.579\pm 0.014$) (see Fig. 3).

Variability in the jamming packing fraction *φ*_J_ has notably been studied for increased frictional interactions between particles, which was linked to a reduction in the packing fraction at jamming^38,56^: if the particles’ contact number is high enough, the jamming transition happens at lower packing fraction for frictional particles than for frictionless particles. The proposed mechanism is the following: increased frictional interactions restrict the reorganization possibilities of the packing, resulting in higher stability of each configuration. In other words, the reduction of collective degrees of freedom available for the granular fabric to reorganize into a denser packing under the applied stress results in a jamming transition happening at a lower packing fraction. Similarly, it is our hypothesis that the lower packing fraction at jamming observed in microgravity results from a decrease in collective degrees of freedom and is manifested in a reduced spatial heterogeneity of the force network.

The absence of the force field due to Earth gravity leads to another related phenomenon: the cohesive inter-particle forces, insignificant when compared to the particles’ weight, become predominant in microgravity. Stronger cohesive interactions also reduce the freedom of motion of each individual particles, hence the granular packing to reorganize into a denser configuration as a whole.

This observation confirms results from one of the first rheology experiments performed in *μ*-g, on board the Space Shuttle^57,58^. A startup curve was measured for a dense (*φ* = 0.65), polydisperse powder, comparing gnd and *μ*-g conditions. The response in *μ*-g was jagged, and samples in *μ*-g exhibited a much higher peak friction strength.

## Discussion

A granular piston-probing experiment was conducted on-ground (gnd) and in microgravity (*μ*-g) (using parabolic flights as a low-gravity platform) to probe the effect of the gravitational acceleration on the compression of a granular medium. We find that the collective behavior of a granular material reveals strong dependence on the gravitational environment.

As the piston rising densifies the granular material above it (region №1), an equivalent empty volume is created below the piston (region №3). Once the material in region №1 reaches a packing sufficiently dense to transmit the load imposed by the piston, a rise of pressure recorded on the container’s walls indicates the formation of a percolated network, and the piston upward motion slows down. Eventually, a jammed state is reached above the piston (region №1): the load imposed by the piston is transmitted to the container’s walls, and eventually the piston stops. On-ground, the fabric of the jammed packing allows for sufficient downward forces to overcome cohesion in creep flow. The formation of an annular heap with a finite angle of repose in region №3 eventually limits the powder rearrangement and jams the piston. In microgravity, the fabric is altered in such a way, that the downward forces are insufficient to overcome cohesion. The piston jams even though region №3 still provides free space.

In our experiments conducted in microgravity, this jammed state is reached while the piston is at lower height, ${h}_{{{{\rm{J}}}}}^{\mu -{{{\rm{g}}}}}\, <\, {h}_{{{{\rm{J}}}}}^{{{{\rm{gnd}}}}}$, than for their repetitions on-ground. We conclude that the packing fraction at jamming is lower in microgravity than on-ground. We support this finding by two physical explanations. Firstly, as the interarticular force balance changes in absence of gravity, cohesive interactions become predominant. Besides, on-ground, particles minimize their potential energy by going to the lower position possible: Earth gravity creates a secondary force field to stimulate reorganization in a different direction to the major compression stress. Under microgravity, such reorganization stimulation does not take place. As a result, in microgravity, powder flow deteriorates; the particles’ configuration is more stable or less likely to reorganize: the packing fraction at jamming is lower in microgravity than on-ground.

In all experimental results presented, the piston height *h* is the parameter measured directly: the *global* packing fraction *φ* is calculated assuming density homogeneity in the container. Local packing fractions are not experimentally accessible in the current setup. We also report a lack of control over the initial state of the granular packing, as the flight time preliminary to the first parabola and repeated hypergravity phases might contribute to creating an initial state specific to the microgravity experiment, that is not reproduced in their on-ground counterpart. To minimize the influence of repeated hypergravity phases on the granular packing, only the first parabolas of each flight are used to calculate *φ*_J_. Besides, the granular samples generally undergo a slight positive acceleration along the *z*-axis at the initial instant of each parabola, which is expected to counteract the compaction caused by hypergravity.

The authors hope that those results will spur interest and further investigation into the influence of gravity on the packing fraction at jamming in granular media. If confirmed, the deterioration of powder flow and lower packing fraction at jamming under reduced gravity could have dramatic consequences for powder handling on sand-covered celestial bodies.

## Methods

### Granular material

**Fig. 8:**
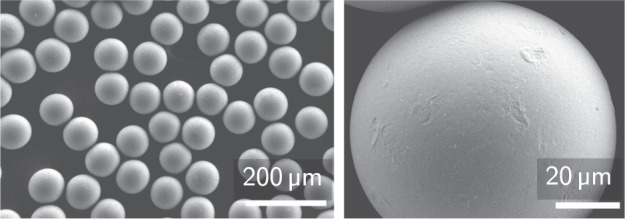
Scanning electron microscopy of the granular material used. The micrographs presented are taken in-house after the completion of the cleaning process.

The granular material used for all experimental results presented is spherical, monodispersed PS powder of mass density *ρ*_*b*_ = 1050 kg m^−3^ and diameter *a* = 80 μm produced by the company *Microbeads* under the name *Dynoseeds*^59^. The powder was washed to ensure a clean, smooth surface, and sieved to avoid outliers (wet-sieving under ultrasound at a frequency of 20 kHz for a duration of 8 hours). The surface state and sphericity of the granular material were verified using scanning electron microscopy. Micrographs before and after the cleaning process were taken, showing the limited roughness and clean surface of the particles after cleaning. Figure 8 shows the cleaned particles, as used in the experiments discussed.

### Bulk modulus of the packing: limits to stresses and speeds

For elastic particles in a given jammed packing, a further reduction of volume, Δ*V*, can be achieved by forcing the particles to deform. Assuming an isotropic compression leads to small typical deformations 〈*ξ*〉/*a* relative to the particle radius *a*, we can estimate the relative volume change as −Δ*V*/*V* ≈ 3〈*ξ*〉/*a*. Such a volume change is related to the pressure Δ*p* = − *K*Δ*V*/*V* by the bulk modulus *K*. Shaebani et al.^60^ have shown that

2 $$K=C\frac{\varphi z}{6\pi a}\times \frac{\partial F}{\partial \xi }(\langle \xi \rangle ),$$

where *z* is the average coordination number, *F*(*ξ*) is the inter-particle force, and *C* is of order one for almost monodisperse packings. Here we assume a nonlinear, Hertzian contact force^61^

3 $$F(\xi )=\frac{\sqrt{2}}{3}\times \frac{{E}_{p}}{1-{\nu }^{2}}{a}^{1/2}{\xi }^{3/2}$$

that is parametrized in terms of *E*_*p*_ and *ν*, Young’s modulus and Poisson’s ratio of the particles’ material, respectively. Using Eq. (3) in Eq. (2), and noting that *φz*/(1−*ν*^2^) ≈ 4 around jamming where *φ* ≈ 0.6, *z* ≈ 6, and for typical Poisson’s ratios *ν* ≈ 0.35, we find

4 $$K\approx \frac{{E}_{p}}{3\pi }\sqrt{2\langle \xi \rangle /a}\approx \frac{{K}_{p}}{\varphi }\sqrt{\langle \xi \rangle /128a},$$

where *K*_*p*_ is the bulk modulus of a particle, and

5 $$\frac{{{\Delta }}p}{E}\approx \frac{\sqrt{2}}{\pi }{\left(\langle \xi \rangle /a\right)}^{3/2}.$$

If we want to limit the deformations of the PS particles (*E*(PS) ≈ 3.3 GPa^62^), to 〈*ξ*〉/*a* ≲ 10^−3^, we need to limit the pressure to Δ*p* ≲ 45 kPa.

With the universal relation *G* = 3*K*/5^60^ for the shear modulus in a packing, the s-wave speed ${c}_{s}=\sqrt{G/\rho }$, can be related to the speed of sound in a particle, ${c}_{p}=\sqrt{3{K}_{p}(1-\nu )/{\rho }_{p}(1+\nu )}$^63^ (*c*_*p*_(PS) ≈ 2350 ms^−1^^62^) by Eq. (4) as:

6 $${c}_{s}\approx {c}_{p}\sqrt{\frac{1+\nu }{5(1-\nu )}}{(\langle \xi \rangle /128a)}^{1/4}\approx 80\,{{{\rm{m}}}}\,{{{{\rm{s}}}}}^{-1},$$

much larger than the piston speed. Note that these are order-of-magnitude estimates only and many quantitative refinements would be possible.

### Mohr-Coulomb material

Following Nedderman^55^, a two-dimensional Mohr–Coulomb material is characterized by three parameters: the macroscopic friction *μ*, the cohesion *c*, and the weight density *γ* = ρ^*^g where ρ^*^ = *ρ*_*b*_*φ* is the mass density of the material at a given packing fraction *φ* (where *ρ*_*b*_ is the density of the particles’ material). The Mohr-Coulomb failure criterion limits the shear stress *τ* for a stable material depending on the normal stress Σ, ∣*τ*∣ ≤ *μ*Σ + *c*. One consequence of this yield criterion is that normal stresses are not isotropic but that there is a finite ratio *k* < 1 between the minor and the major principal normal stress, the Janssen constant.

Assuming that the major and minor principal stress axes are aligned with the vertical and horizontal orientation of the container, one obtains the vertical force balance^50,55^

7 $$\frac{d{{{\Sigma }}}_{zz}}{dz}\pm \frac{{{{\Sigma }}}_{zz}}{\lambda }=\gamma$$

and the boundary condition Σ_*zz*_(*h*) = *σ* for an external stress *σ* applied at height *z* = *h*. The sign between the first and the second term depend on the direction of the force. The length scale *λ*, associated with the screening of forces described by Eq. (7), depends on the geometry of the problem. For a major principal stress in the vertical orientation (the classical Janssen analysis), *λ* ∝ 1/*k*, whereas for a horizontal major stress, *λ* ∝ *k*. For a container of width *D* and no external stress, Janssen^50^ obtained for the horizontal stress

8 $${{{\Sigma }}}_{rr}(z)=\frac{\gamma D}{4\mu }\left(1-{e}^{-4\mu kz/D}\right)$$

and for the vertical stress Σ_*zz*_(*z*) = Σ_*rr*_(*z*)/*k*.

### Data processing

**Fig. 9:**
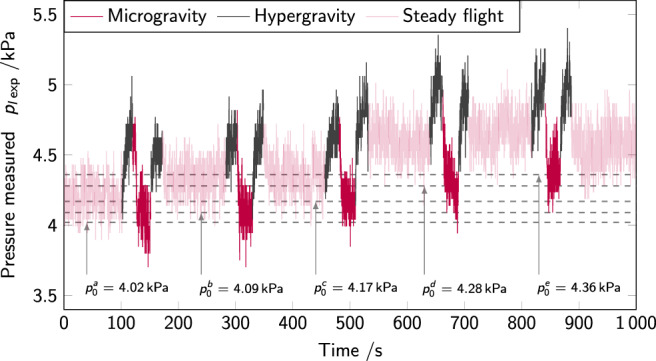
Pressure ${p}_{\exp }$ measured on the bottom sensor inside an experimental cell filled with powder but with no motion of the piston. Pressure changes are solely due to changes in acceleration during parabolic flight maneuvers. The offset pressures found from the microgravity phases are shown as dashed lines, labeled ${p}_{0}^{a,\,b,\,c,\,d,\,e}$. The increase in offset pressure throughout the experiments shown here is clearly visible, amounting to +0.34 kPa in 1000 s.

The pressure sensors placed inside the container were observed to offset due to the constant pressure applied on the sensors by the granular packing, once the material is placed inside the container. This offset was found to be ~4–5 kPa on all experiments (both on-ground and in microgravity). However, this offset pressure is not constant, but increases with the experiment’s duration, rendering exact quantitative pressure measurement challenging. This shift can be observed in Fig. 9, where the raw pressure measured ${p}_{\exp }$ is shown: throughout the five parabolas described by the Zero-g plane in the course of 1000 s, the pressure increases by approximately + 0.34 kPa.

In order to retrieve the pressure evolution *p*, from the pressure measured ${p}_{\exp }=p+{p}_{0}$, the offset pressure *p*_0_ was calculated per experiment (per parabola in the case of microgravity experiments).

For the microgravity experiments, *p*_0_ is deducted from the steady-state pressure (once a relatively stable value is reached after transient decrease): under reduced gravitational acceleration and before the start of piston rise (i.e., at *t* = 0 s), the expected pressure is *p*(*t* = 0 s) = 0 Pa, as no pressure is applied on the sensors. Therefore, we assume that the remaining pressure measured equals the offset *p*_0_, and use this value to estimate $p={p}_{\exp }-{p}_{0}$.

For the measurements conducted on-ground, the reference value used is the pressure at the beginning of the experiment, before the piston starts rising. At this point, the pressure recorded should be solely due to the weight of the powder inside the container: using the mass of the powder at the sensor’s height, we calculate the expected horizontal pressure *p*_*h*_ ≡ Σ_*rr*_(*h*) from Eq. (8). Numerical parameters used are in Table 2. The offset pressure ${p}_{0}={p}_{\exp }-{p}_{h}$ is calculated for each experiment to obtain *p*.

**Table 2:** Numerical values of the parameters describing our system: a polystyrene powder in a polycarbonate container.

	Parameter	Unit	Value	Absolute uncertainty
*φ*	packing fraction (initial)	–	0.55	–
*D*	container’s diameter	m	5.40 × 10^−2^	10^−4^
*d*	piston’s diameter	m	3.60 × 10^−2^	10^−4^
*m*	mass of powder	kg	5.00 × 10^−2^	10^−4^
*ρ*_*b*_	density in bulk	kg m^−3^	1050	10
ρ*	powder density	kg m^−3^	580	10
*g*	gravitational constant	ms^−2^	9.81	–
*μ*	static friction coefficient	–	0.3	–
*k*	Janssen coefficient	–	0.4	–

A plethora of ways are available to estimate the Janssen coefficient *k* ∈ [0, 1]. Here, we follow the recommendations given in ref. ^65^. The PS properties are taken from the powder manufacturer data sheet^59^. The powder density is defined as ρ* = *ρ*_*b*_ *φ*, PS density in bulk times the powder packing fraction.

### Formal uncertainty

Formal uncertainty due to measurement limitations is calculated for the packing fraction from the mass *m* of granulate, the density *ρ*_*b*_ of the particles’ material (in bulk), and the volume occupied by the granular material, which depends on the diameter *D* squared and height *h* of the piston. As each variable is measured independently, we rely on propagation of uncertainty laws and calculate the relative error *δφ*/*φ* as the sum of relative errors on each variable.

The absolute uncertainties for each measured variable are given in Table 2. The height *h* of the piston is measured from image analysis using a scale placed right behind the piston’s axis, which results in an absolute uncertainty of *δh* = 0.5 mm.

The resulting absolute uncertainties *δφ* are plotted as error bars in Fig. 3. The maximum absolute uncertainty *δφ*^gnd, μ−g^ = 0.027, which represents a relative error of 5%, is taken as accuracy measurement for the average ${\varphi }_{J}^{{{{\rm{gnd}}}}}$ and ${\varphi }_{J}^{\mu -{{{\rm{g}}}}}$.

### Statistical significance

To validate the claim that the gravitational environment influences the packing fraction at jamming measured in our system, a statistical significance test is essential. The test is done on the two series of four values for ${\varphi }_{J}^{{{{\rm{gnd}}}}}$ and ${\varphi }_{J}^{\mu -{{{\rm{g}}}}}$ given in Fig. 3. The null hypothesis is that the cumulative distributions are identical, $P({\varphi }_{J}^{{{{\rm{gnd}}}}} < {\varphi }_{J})=P({\varphi }_{J}^{\mu -{{{\rm{g}}}}} < {\varphi }_{J})$; the alternative hypothesis is that $P({\varphi }_{J}^{{{{\rm{gnd}}}}} < {\varphi }_{J}) < P({\varphi }_{J}^{\mu -{{{\rm{g}}}}} < {\varphi }_{J})$.

All values given for packing fraction at jamming are calculated solely from the experiment conducted on the first parabola of each flight, before any powder has flowed under the piston (region no. 1 in Fig. 7), as shown in Fig. 2a–e. Therefore, only four repetitions of the experiment are available. For small samples where normal distribution cannot be assumed, a nonparametric statistical significance test is preferable. We use the sum of ranks test known as Mann–Whitney *U* test^64^.

We find a probability of the null hypothesis of only 1.4%^64^. We can therefore confidently conclude that for the limited amount of data available, the probability of the distributions of ${\varphi }_{J}^{{{{\rm{gnd}}}}}$ and ${\varphi }_{J}^{\mu -{{{\rm{g}}}}}$ being equal is low enough to be rejected, which supports our conclusion that the packing densities at jamming are different between ground and microgravity.

### Reporting summary

Further information on research design is available in the Nature Research Reporting Summary linked to this article.

## Supplementary information

Supplementary Material

Reporting Summary

## Data Availability

The datasets used in this article are publicly available in the Zenodo repository 7101542.

## Code availability

The codes used to generate the plots are available from the corresponding author upon reasonable request.
