# Supplementary material for: Granular piston-probing in microgravity: powder compression, from densification to jamming
Source: NPJ Microgravity. 2022 Nov 5;8:48. doi: 10.1038/s41526-022-00235-2 (PMC9637118; doi:10.1038/s41526-022-00235-2)
Supplement: Supplementary file 1 — Supplementary Material [file 41526_2022_235_MOESM1_ESM.pdf]

Supplementary information

Granular piston-probing in microgravity: powder compression, from densification to jamming

Olfa D'Angelo*[1,2], Anabelle Horb†[3], Aidan Cowley[3], Matthias Sperl[1,4], and W. Till Kranz[4,1]

[1] *Institut für Materialphysik im Weltraum, Deutsches Zentrum für Luft- und Raumfahrt (DLR), 51170 Köln, Germany.*
[2] *Institute for Multiscale Simulation, Universität Erlangen-Nürnberg, Cauerstraße 3, 91058 Erlangen, Germany.*
[3] *European Astronaut Centre (EAC), European Space Agency (ESA), 51170 Köln, Germany.*
[4] *Institut für Theoretische Physik, Universität zu Köln, 50937 Köln, Germany.*

QUASI-2D TOY MODEL

Using stress-birefringent particles, force chains can be made apparent in granular media [1, 2]. To have an insight into the mesoscopic-scale mechanisms at stake in our piston-probing experiment, it is reproduced in quasi-two dimensional (2D) using a bidispersed packing of stress-birefringent disc-particles. This setup is used to obtain a direct observation of the differences in force chains formation due to the present or absence of a secondary force field created by Earth gravitational acceleration on the particles.

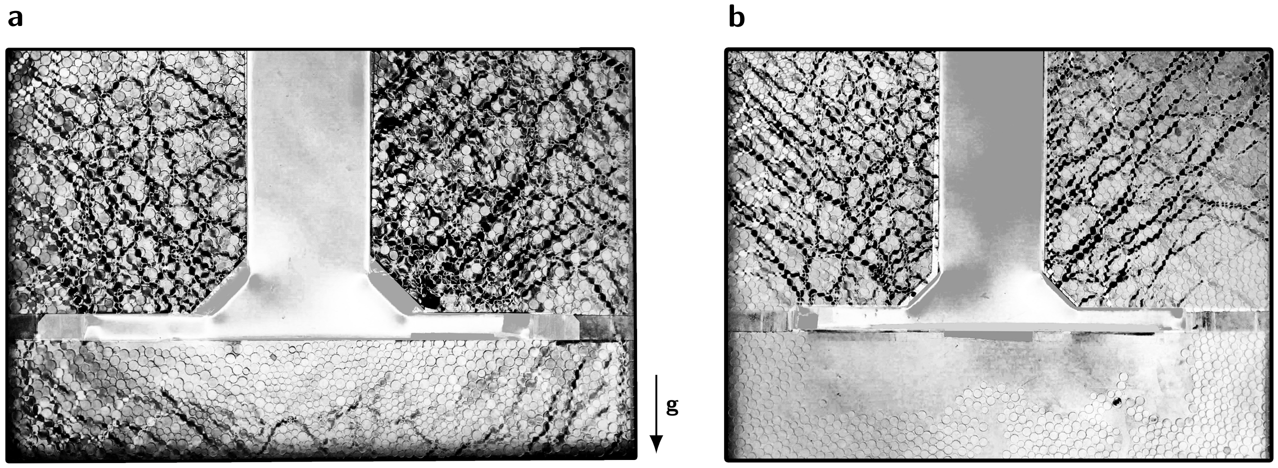

Vertical, analogous to ground

Horizontal, analogous to microgravity

Supplementary Figure 1: Force chains shown by stress-birefringent particles in an equivalent piston-probing setup in 2D. Force chains are shown in black, while unloaded particles are shown in white. The different gravitational environments are reproduced by placing the setup (a) vertical and (b) horizontal, respectively reproducing the presence of a secondary force field due to Earth gravity, and its absence, as found in weightlessness. Photographs were taken by the authors; colors have been modified to highlight the force chains in dark while keeping the particles visible.

Images resulting from this quasi-2D reproduction of the piston-probing setup are presented in Supplementary Figure 1. The gravitational forces were reproduced by placing the quasi-2D experimental box normal to the ground for Supplementary Figure 1(a), hence using the downward force due to gravity to draw all particles downward vertically. For reproducing the microgravity experiment (Supplementary Figure 1(b)), the experimental box was placed horizontally, parallel to the ground: in this position, no force leads the particles towards the bottom of the container.

Comparing of the two images, the regions proposed in Figure 7 of the article becomes visible, as well as the effect of the secondary force field for the particles under the piston. For both images, above the piston a large

*Corresponding author: olfa.dangelo@mail.com
†Current address: Omnidea, Lda. – Polypark, Nùcleo Empresarial da Arruda dos Vinhos, Estrada da Quinta de Matos 4, 2630-179 Arruda dos Vinhos, Portugal.

number of force chains appear under compression of the rising piston. Under the piston however, on-ground (gnd) (Supplementary Fig. 1(a)) force chains are visible close to the container wall, where the weight of the granular packing placed on the upper part of the container is passed down onto the bottom particles. Under this pressure, as well as the weight of the particles themselves, the packing crumbles under the platform and the volume emptied by the piston rise is filled intermittently. On the other hand, in microgravity (μ-g) (Supplementary Figure 1(b)), under the piston's platform no force chains are visible, and the space created by the piston rise is left empty.

SUMMARY OF EXPERIMENTS PRESENTED

All experiments conducted to collect the data presented are summarized in Supplementary Table 1.

Supplementary Table 1: Summary of experiments reported throughout the article.

Reference(s) in article	Granular material used	Parabola number	Initial position of piston	Environment (acceleration)
Fig. 3: Exp. 1	polystyrene (PS) powder, ⌀ 80 µm	–	bottom of the cell	on-ground ($g = 9.81\,\mathrm{m\,s^{-2}}$)
Fig. 3: Exp. 2	PS powder, ⌀ 80 µm	–	bottom of the cell	on-ground ($g = 9.81\,\mathrm{m\,s^{-2}}$)
Fig. 3: Exp. 3	PS powder, ⌀ 80 µm	–	bottom of the cell	on-ground ($g = 9.81\,\mathrm{m\,s^{-2}}$)
Fig. 3: Exp. 4, Fig. 2(gnd), Fig. 5(a)	PS powder, ⌀ 80 µm	–	bottom of the cell	on-ground ($g = 9.81\,\mathrm{m\,s^{-2}}$)
Fig. 4(a)	PS powder, ⌀ 80 µm	–	half-height of the cell	on-ground ($g = 9.81\,\mathrm{m\,s^{-2}}$)
Fig. 3: Exp. 5	PS powder, ⌀ 80 µm	parabola 0 (first of flight day)	bottom of the cell	on parabolic flight ($g \approx 10^{-2}\,\mathrm{m\,s^{-2}}$)
Fig. 3: Exp. 6 Fig. 5(b)	PS powder, ⌀ 80 µm	parabola 0 (first of flight day)	bottom of the cell	on parabolic flight ($g \approx 10^{-2}\,\mathrm{m\,s^{-2}}$)
Fig. 3: Exp. 7 Fig. 2 (μ-g)	PS powder, ⌀ 80 µm	parabola 0 (first of flight day)	bottom of the cell	on parabolic flight ($g \approx 10^{-2}\,\mathrm{m\,s^{-2}}$)
Fig. 3: Exp. 8	PS powder, ⌀ 80 µm	parabola 0 (first of flight day)	bottom of the cell	on parabolic flight ($g \approx 10^{-2}\,\mathrm{m\,s^{-2}}$)
Fig. 4(b)	PS powder, ⌀ 80 µm	parabola 0 (first of flight day)	half-height of the cell	on parabolic flight ($g \approx 10^{-2}\,\mathrm{m\,s^{-2}}$)
Fig. 6, Fig. 9	PS powder, ⌀ 80 µm	parabola 2 (Fig. 6), parabolas 1 to 5 (Fig. 9)	bottom of the cell (no motion)	on parabolic flight, including steady flight, hypergravity and low gravity periods

SUPPLEMENTARY REFERENCES

[1] A. Abed Zadeh, J. Barés, T. A. Brzinski, K. E. Daniels, J. Dijksman, N. Docquier, H. O. Everitt, J. E. Kollmer, O. Lantsoght, D. Wang, M. Workamp, Y. Zhao, and H. Zheng. Enlightening force chains: a review of photoelasticimetry in granular matter. *Granular Matter*, 21:83, 2019. doi: 10.1007/s10035-019-0942-2.

[2] K. E. Daniels, J. E. Kollmer, and J. G. Puckett. Photoelastic force measurements in granular materials. *Review of Scientific Instruments*, 88(5):051808, 2017. doi: 10.1063/1.4983049.
